# Supplementary material for: Defining and quantifying fatigue in the rugby codes
Source: PLoS One. 2023 Mar 10;18(3):e0282390. doi: 10.1371/journal.pone.0282390 (PMC10004502; doi:10.1371/journal.pone.0282390)
Supplement: S1 Fig — Responses to questions A) “In the context of your sport (i.e. rugby code), how do you (or would you) currently define player fatigue?”, and B) “In the context of your sport (i.e. rugby code), which specific ‘types’ of fatigue (e.g. neuromuscular, metabolic, perceptual, biochemical, autonomic etc.) do you consider it important to monitor (please specify more than one ‘type’ if it is applicable)?” presented as 30 word clouds which are the 30 most commonly used words and phrases used in the responses in round one SMEs (n = 42). (DOCX) [file pone.0282390.s001.docx]

Supplementary Figure 1


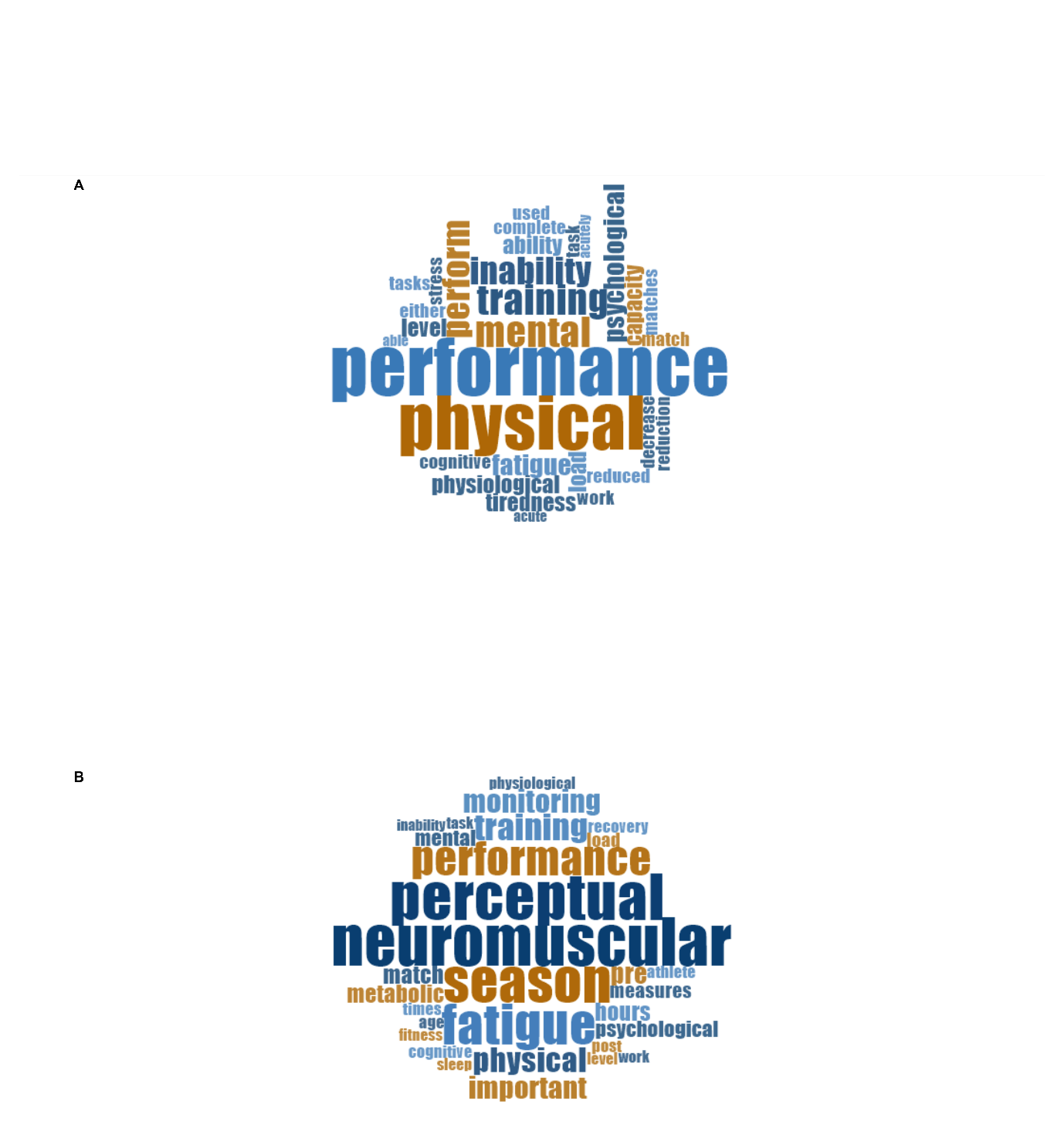


**S1 Fig 1**. Responses to questions A) “In the context of your sport (i.e. rugby code), how do you (or would you) currently define player fatigue?”, and B) “In the context of your sport (i.e. rugby code), which specific ‘types’ of fatigue (e.g. neuromuscular, metabolic, perceptual, biochemical, autonomic etc.) do you consider it important to monitor (please specify more than one ‘type’ if it is applicable)?” presented as 30 word clouds which are the 30 most commonly used words and phrases used in the responses in round one SMEs (n = 42).
