# Supplementary material for: Defining and quantifying fatigue in the rugby codes
Source: PLoS One. 2023 Mar 10;18(3):e0282390. doi: 10.1371/journal.pone.0282390 (PMC10004502; doi:10.1371/journal.pone.0282390)
Supplement: S1 Table — (DOCX) [file pone.0282390.s002.docx]

**S1 Table 1**. Characteristics of items which did not achieve consensus (≥67% agreement) across two adjacent categories in the two round Delphi study including the largest consensus agreement obtained, the median rating, and the round in which agreement and rating was obtained.

| **Domain** | **Item** | **Feature (Importance/Feasibility)** | **Largest Consensus Agreement (%)** | **Median Rating (1-5)** |
| --- | --- | --- | --- | --- |
| **Round One** | | | | |
| Neuromuscular Performance | Countermovement jump – height (cm) and flight time (sec) | Importance | 63.6 | 3.0 |
|  | Plyometric pushup – force (N) and power (W) | Feasibility | 65.0 | 3.0 |
|  | Plyometric pushup - height (cm/m) and flight time (s) | Feasibility | 55.0 | 3.0 |
|  | Countermovement jump - MyJump/MyJump2 jump height (cm) | Feasibility | 55.6 | 3.0 |
| Cardio-autonomic | Heart-rate variability (HRV) – taken at rest | Importance | 63.6 | 3.0 |
|  | Heart-rate (bpm) - during standardised sub-maximal exercise | Feasibility | 63.6 | 4.0 |
|  | Heart-rate (bpm) - recovery post-exercise | Feasibility | 63.6 | 3.0 |
| **Round Two** | | | | |
| Neuromuscular Performance | Neck - isometric force (N) | Importance | 60.9 | 2.0 |
|  | Neck - isometric force (N) | Feasibility | 60.9 | 2.0 |
|  | Groin squeeze isometric force (N) | Feasibility | 56.5 | 4.0 |
|  | Running mechanics - assessed qualitatively | Feasibility | 56.5 | 2.0 |
|  | Countermovement jump - MyJump/MyJump2 jump height (cm) | Feasibility | 65.2 | 4.0 |
| Cardio-autonomic | Resting heart-rate - without prior exertion (bpm) | Importance | 56.5 | 3.0 |
|  | Resting heart-rate - without prior exertion (bpm) | Feasibility | 60.9 | 4.0 |
|  | Heart-rate (bpm) - during standardised sub-maximal exercise | Feasibility | 60.9 | 3.0 |
|  | Heart-rate (bpm) - recovery post-exercise | Feasibility | 56.5 | 3.0 |
| Self-report | Sleep quantity | Feasibility | 56.5 | 4.0 |
|  | Motivation | Feasibility | 52.2 | 3.0 |
