# Supplementary material for: Defining and quantifying fatigue in the rugby codes
Source: PLoS One. 2023 Mar 10;18(3):e0282390. doi: 10.1371/journal.pone.0282390 (PMC10004502; doi:10.1371/journal.pone.0282390)
Supplement: Attachment — Submitted filename: Response to Reviewer Comments_Final_PLOS_Clean.docx [file pone.0282390.s003.docx]

| Reviewer Comment | **Author Response** |
| --- | --- |
| Reviewer #1: The study aimed to develop a definition of fatigue in rugby and determine which methods and metrics are important and feasible to implement post-match fatigue. The study is interesting and well written. The issue presents substantial practical application to monitoring fatigue and recovery of rugby athletes. One concerns is about fatigue definition. Through the manuscript authors claimed to define fatigue, I suggest to include “define fatigue IN RUGBY” (as presented in some parts) in all manuscript. Other minor concerns are presented below. | The authors thank the reviewer for their suggestion. “in rugby” has been added throughout the document to defining fatigue. The numerous locations where this has occurred are highlighted through TrackChanges on the document. |
| Abstract The abstract must be improved. The results of the manuscript should be presented in the abstract (which variable were selected in the two rounds, fatigue in rugby definition). | The authors agree with this suggestion. The revised abstract now includes the fatigue definition and examples of the highly rated methods and metrics – Lines 39 – 48 “*This definition then obtained 96% agreement from the SME in round two. The definition was that fatigue in rugby refers to a reduction in performance-related task ability which is underpinned by time-dependent negative changes within and between cognitive, neuromuscular, perceptual, physiological, emotional, and technical/tactical domains. Further, 33 of the 55 items in the neuromuscular performance, cardio-autonomic, or self-report domains achieved consensus for importance and/or feasibility to implement. From these items, highly rated methods and metrics included countermovement jump force/power (neuromuscular performance), heart rate variability (cardio-autonomic measures), and soreness, mood, stress, and sleep quality (self-reported assessments)*.” |
| Introduction In my point of view the first paragraph could be smaller only indicating the importance of quantifying external and internal load of training and match play. | The authors agree with this suggestion and have removed content from this first paragraph. The revised paragraph now reads - Lines 59-66 - “*The rugby codes (i.e., rugby union, rugby league, rugby sevens [hereafter termed ‘rugby’]) are team-sports that include multiple complex physical, perceptual, and technical demands on players. For rugby, there are multiple methods and metrics to monitor each specific component of training and match-play through technologies such as global positioning systems (GPS), accelerometers, and heart-rate telemetry to quantify both the output of the player on the field (e.g. frequency, intensity, and duration of locomotor and collision actions – termed the external load), and the players psycho-physiological responses (e.g., heart-rate, blood lactate concentration, rating of perceived exertion [RPE] - termed the internal load) to the completed external loads [1-3]*” |
| Methods  1)How many subjects were recruited from social media, professional networks and email? Can this be considered a limitation of the present study?  2)The countermovement jump (force and power) was considered the value evaluated by force plate or estimated by jump heigh?  3)Was the variable inserted in round 2 in both situations (importance and feasible) when consensus was found in one or another (importance or feasible) during round 1? If so, why? | 1) All the participants were recruited initially through these different online communication methods. This is consistent with recruitment strategies in prior Delphi studies in sport and other domains – Lines 149-150 – “*This is consistent with participant recruitment strategies in prior Delphi studies in sport and other domains [20, 21, 26].*” Further, due to the COVID 19 pandemic and related restrictions, it was not possible or ethically appropriate to undertake recruitment or data collection via in person methods. The authors agree that this could have been a limitation and this has been indicated in the limitations section, Lines 425 – 428 – “ *Further, only SME who responded to being contacted via social media, professional networks, and Email were included, and this could be a limitation for the sample (e.g., the types of SME who would respond to these kind of invitations).*”  2) The technique used was considered as it is measured directly from force plates/platforms and not estimated through equations from height/flight time. This has been included in the revised text – Lines 170 - 171 – “… *indicating force and power metrics in the countermovement jump test measured directly through force plates/platforms.*”  3) The item was only included in round two for the component (importance and feasibility to implement) that did not achieve consensus in round one. The authors agree that this could be clearer and have revised the text accordingly – Lines 200 – 203 - “ *If there was consensus for an item for importance or feasibility to implement but not for the other component (e.g., for importance but not for feasibility to implement, or vice versa) in round one, it was only included in round two for the component for which there was not consensus.”* |
| Results Please, can you describe better “others” (what would be Olympic sport)? Page 13 line 273: review “WHICH IS ARE…” | The authors agree with this suggestion, and the revised text indicates this - Lines 160-162 – “*When SME held roles at other levels of professionalism (e.g., Olympic-level) or in other sports (e.g., track and field), this was categorised as “other” in the results*.“, and this is also included with the revised figure legend for Figure 1 including the examples. |
| Discussion  Would be better to perform a third round with fatigue definition (with other group of participants)? | The authors agree that refining the fatigue specific definition with a further round of the Delphi could have potentially been of use. However, given the decrease in the number of participants from round one to round two, it was expected that the number of participants would have decreased further for any future rounds which would have further limited the amount of data produced. This loss of sample size would also have potentially had an impact on the strength of recommendations that would have been able to be garnered from the SME and then integrated into an updated definition. Further, we discuss the lack of a third round as a potential limitation in the limitations section – Lines 439 – 441 – “*Further, although the definition achieved a high amount of agreement (96%), the SME did not have the option to provide suggested changes to the fatigue definition in round two.*” The authors agree that further refinement of the definition is necessary to identify the relevant determinants of fatigue in rugby and state – Lines 38 – 391 – “*Further research can refine this agreed upon definition, as necessary, and determine the relative contributions of the presented components of fatigue (and their interactions) in rugby.*” |

| Reviewer #2: The study aimed to develop a definition of fatigue in rugby, and to determine the agreement with the definition of fatigue. Also, it aimed to outline which methods and metrics the support staffs consider important and feasible to implement and quantify post-match fatigue. Experts undertook the Delphi questionnaire twice, the first to define fatigue and further the consensus of 33 items on neuromuscular performance, cardio-autonomic, or self-report domains achieved consensus for importance and feasibility. The authors conclude that using two rounds of a questionnaire with a modified Delphi study, a consensus was obtained on a fatigue definition for rugby. They define fatigue as a reduction in performance-related task ability, which is underpinned by time-dependent negative homeostatic changes within and between cognitive, neuromuscular, perceptual, physiological, emotional, and technical/tactical domains. |  |
| --- | --- |
| I would like to congratulate the authors for this interesting study. It is mostly well-written in the introduction and discussion, the methods are detailed, and the figures are sharp and clear. Although, there are some points that should be addressed.  1) Why only post-match fatigue? It is clear measuring fatigue in before, the day, and after match is crucial and this choice is not well explained in the text.  2) The introduction repeats some information, for example, citing methods to quantify external and internal loads. Also, several lines of thinking are raised in each paragraph, which makes them huge and tiresome. In my humble opinion, the authors could write about a specific issue in each paragraph and avoid writing more than 15 lines for each of them.  3) In addition, the gap of the study should be improved. What’s the real difference between rugby and most of other sports that rugby would need a different definition of fatigue? The authors should mainly focus on that to convince the readers.  4) Also, in the literature there are many fatigue definitions, including some really similar to the authors’. So, it should be better explained and compared this study definition of fatigue with the ones available in the literature.  5) The same should be applied in the discussion session, there’s a paragraph with 30 lines and it turns the reading really exhausting. Also, I recommend the authors to in each paragraph write about how that result that is being discussed is modifying the actual literature. | The authors thank Reviewer 2 for their comments. Each of these relates to a different point so they have been numbered and separated below to respond to them accordingly.  1) It is agreed that it is important to quantify the players fatigue state, at multiple times (such as pre- and post-match), particularly in the in-season period, to ensure players are returning to their individual baselines. The relevant text in the introduction section indicates this – Lines 75 – 78 – “*Pre- and post-match monitoring of each player’s fatigue state is therefore necessary in the in-season period to identify if players are or are not returning to their individual baseline state between matches* *(with multiple post-match testing time points advisable)* *[9].*” Failure to return to an individual baseline may require the application of extra recovery interventions or reflect a chronic issue such as overtraining. During the pre-season period short term changes in fatigue are likely less crucial to consider as they may indicate adaptations in physical fitness (i.e., the fitness-fatigue relationship [Banister]).  2) The authors have removed content throughout the Introduction section which ensures each paragraph (with the exception of one 16 line paragraph) meets the 15 line limit indicated by Reviewer 2. The authors believe that the Introduction section states the complex nature of fatigue and fatigue monitoring in rugby. This necessitates introducing rugby (paragraph 1), fatigue as a construct and it’s quantification (paragraph 2), how fatigue is currently defined in rugby and the limitations of these definitions (paragraph 3), current literature on fatigue monitoring (paragraph 4), introduction to Delphi methods (paragraph 5), and the rationale for the current study (paragraph 6). The content in these paragraphs is necessary to provide the reader with the background knowledge on which the present study was designed and conducted. Where possible the authors have removed unnecessary text in the introduction section.  The authors believe that the rugby football codes are distinct from other sports which have received fatigue definitions primarily due to the frequency and intensity of collisions and locomotor actions. This has been stated - Lines 85 – 869 “…*context of the unique demands of rugby (i.e., the frequency and intensity of collisions and locomotor actions) [14].*” The authors also state the differences between previous definitions of fatigue in rugby in the text - Lines 92 – 94 – “*Previously proposed operational definitions of fatigue in rugby have been taken from prolonged endurance cycling exercise [16], which is a different exercise mode with different external and internal loads to that of rugby.*”  The authors are not aware of the definitions that have specific relevance to rugby or this article that Reviewer 2 is referring to, which are not already stated within the manuscript (e.g., Lines 92 - 94).  The authors agree with this suggestion. The authors have also had to balance this comment from Reviewer 2 requesting to remove content and streamline the discussion.  In line with this comment and the previous requests, the authors have revised the paragraph in the discussion that the reviewer indicates and removed unnecessary content throughout the discussion to facilitate reading and understanding both for the reader and Reviewer Two. The results of the current study add to the literature in multiple ways, such as the development of a definition of fatigue in rugby, which is presently lacking in the literature. This is discussed – Lines 38 – 404.  The authors also provide an example of how this definition can be refined and developed in the future – Lines 387 – 391 – “*In the present study the SME agreed upon a definition which includes performance, perceptual, physiological, emotional, and cognitive components [7, 41, 46]. Further research can refine this agreed upon definition, as necessary, and determine the relative contributions of the presented components of fatigue (and their interactions) in rugby*”. The authors believe these examples provide the necessary context to the results of the present study and how they add to the current literature and could be used in future research. Finally, the authors have included a practical applications section of how the results of the present study could be used by practitioners – Lines 462 – 488. |
