# Supplementary material for: Defining and quantifying fatigue in the rugby codes
Source: PLoS One. 2023 Mar 10;18(3):e0282390. doi: 10.1371/journal.pone.0282390 (PMC10004502; doi:10.1371/journal.pone.0282390)
Supplement: Attachment — Submitted filename: Response to Reviewer Comments Round 2_v2_Submit.docx [file pone.0282390.s004.docx]

Reviewer #1: The study entitled “Defining fatigue and quantifying post-match fatigue in the rugby codes” aimed to set up a definition of fatigue which could fit for a range of Rugby modalities, as well as determine the methods and metrics for fatigue assessment which experts consider important in the rugby daily-based routine.
I would like to congratulate the authors for the study which is really interesting and highly applicable for coaches, sports scientists, performance analysts, practitioners, academic researchers etc. Follow bellow minor details and few suggestions.

**The authors wish to thank the reviewer for reviewing the manuscript and their recommendations and suggestions. The responses to each of these is presented below with the authors responses in bold.**

1) The first paragraph of the introductions is confusing, most likely due the excessive information inside the parentheses. Please, revise this paragraph.

**This paragraph has been revised from Line 62 to Line 64 – 65 of the updated text and the text has been amended to remove the parentheses which fixes the issue that the Reviewer indicates.**

2) The second paragraph of introduction (from line 65 to 82) and the sixth paragraph of the discussion (from line 423 to 448) are too long, please, split each one in two paragraphs.

**These paragraphs have been revised and each split in to two paragraphs where it was most appropriate to do so, based on the content of these paragraphs.**

3) I would suggest to the author to add a figure of the experimental design timeline. Despite well explained, the many steps of the study would be better visualized by the readers with a figure of the time domain and relevant information (e.g. sample size of each round).

**Figure 1 is now a flow chart which details the timeline of the study, including recruitment information, content of the questionnaire in each round, and the number of participants.**

4) There are several spots in the text with typo errors (lines 271, 272, 283, 355), please double check throughout the manuscript.

**The authors have checked the manuscript for typological and grammatical errors and revised these (see manuscript). The authors have used the PLOS One guidelines (**[**https://journals.plos.org/plosone/s/criteria-for-publication#loc-5**](https://journals.plos.org/plosone/s/criteria-for-publication#loc-5)**) on using Standard English and therefore words such as “analyse” and “specialise” are used instead of “analyze” or “specialize” as would be the case for American English.**

5) I’m not sure if I missed that in the supplementary files, however, would be interesting provide the questionnaires in their final form.

**The authors agree however, due to the length of the questionnaire document, a supplementary file would not be appropriate. The survey content has been uploaded to a public open access repository, the Open Science framework (**[**https://osf.io/p7vud/**](https://osf.io/p7vud/)**). This information has been included in the text (Line 184 of the updated text).**

6) What was considered “dummy responses”? (line 245)

**A response was considered a dummy response if the respondent, for example, responded with an incoherent or one letter or word responses before moving on to the next question. These examples have been included in the text as examples.**

7) Finally, two points to the authors think about.
First, despite the descriptive nature of the study, would be interesting provide some kind of sample size calculation and/or statistical power. That would strengthen the paper.

**Sample sizes calculations to determine statistical power are generally not considered as part of Delphi studies (Trevelyan and Robinson, 2015) and samples can vary widely (e.g., 6 – 50 participants [Birko et al., 2015]). Importantly, as there are no statistical comparisons between the outcome measures (e.g., null-hypothesis significance testing, p values, effect sizes), determining statistical power to detect such differences is not relevant for this study. Whilst an increase of the size of the sample will increase generalisability of the resulting conclusions, the Delphi method requires a qualitative approach to the sample which relies on recruiting the subject matter experts in a given area. The authors believe we have achieved this by the targeted recruitment (i.e., directed emails, use of professional networks) and the inclusion/exclusion criteria. This ensured that the sample is homogenous with respect to their expertise, a recommendation of Trevelyan and Robinson (2015), and that the study has measured the consensus among these subject matter experts with a sample size which is comparable to other Delphi studies in sport.**

8) Second, I don’t agree with “post-match fatigue quantification”. According to the first open-ended question, MD-2 is the second most frequent condition of fatigue assessment. That would not be a “pre-match fatigue” depend on the day of prior match? I suggest (even in the manuscript title) “quantifying match fatigue” only.

**The authors agree with this suggestion, and this has been revised throughout the manuscript with the removal of “post-match” in the context of fatigue.**

**References**

Birko, S., Dove, E. S., & Özdemir, V. (2015). Evaluation of Nine Consensus Indices in Delphi Foresight Research and Their Dependency on Delphi Survey Characteristics: A Simulation Study and Debate on Delphi Design and Interpretation. PloS one, 10(8), e0135162.

Trevelyan, E. G., & Robinson, N. (2015). Delphi methodology in health research: how to do it? European Journal of Integrative Medicine, 7(4), 423-428.
